# Analysis of Dysferlin Direct Interactions with Putative Repair Proteins Links Apoptotic Signaling to Ca^2+^ Elevation via PDCD6 and FKBP8

**DOI:** 10.3390/ijms24054707

**Published:** 2023-02-28

**Authors:** Dennis G. Drescher, Marian J. Drescher, Dakshnamurthy Selvakumar, Neeraja P. Annam

**Affiliations:** 1 Laboratory of Bio-otology, Department of Otolaryngology, Wayne State University School of Medicine, Detroit, MI 48201, USA; 2 Department of Biochemistry, Microbiology and Immunology, Wayne State University School of Medicine, Detroit, MI 48201, USA

**Keywords:** calcium dependence, C2 domains, dysferlin, FKBP8, limb girdle muscular dystrophy type 2B/R2, PDCD6, protein–protein interactions, surface plasmon resonance

## Abstract

Quantitative surface plasmon resonance (SPR) was utilized to determine binding strength and calcium dependence of direct interactions between dysferlin and proteins likely to mediate skeletal muscle repair, interrupted in limb girdle muscular dystrophy type 2B/R2. Dysferlin canonical C2A (cC2A) and C2F/G domains directly interacted with annexin A1, calpain-3, caveolin-3, affixin, AHNAK1, syntaxin-4, and mitsugumin-53, with cC2A the primary target and C2F lesser involved, overall demonstrating positive calcium dependence. Dysferlin C2 pairings alone showed negative calcium dependence in almost all cases. Like otoferlin, dysferlin directly interacted via its carboxy terminus with FKBP8, an anti-apoptotic outer mitochondrial membrane protein, and via its C2DE domain with apoptosis-linked gene (ALG-2/PDCD6), linking anti-apoptosis with apoptosis. Confocal Z-stack immunofluorescence confirmed co-compartmentalization of PDCD6 and FKBP8 at the sarcolemmal membrane. Our evidence supports the hypothesis that prior to injury, dysferlin C2 domains self-interact and give rise to a folded, compact structure as indicated for otoferlin. With elevation of intracellular Ca^2+^ in injury, dysferlin would unfold and expose the cC2A domain for interaction with annexin A1, calpain-3, mitsugumin 53, affixin, and caveolin-3, and dysferlin would realign from its interactions with PDCD6 at basal calcium levels to interact strongly with FKBP8, an intramolecular rearrangement facilitating membrane repair.

## 1. Introduction

As a critical component of a membrane repair complex, dysferlin is hypothesized to orchestrate sealing of membrane defects in muscles, with dysferlin mutation and consequent dysfunction giving rise to limb girdle muscular dystrophy type 2B (also called LGMD R2 dysferlin-related [1]). Patients afflicted with LGMD2B are limited in their ability to repair muscle lesions. Previous studies have indicated that the annexins, calpain, mitsugumin, affixin, caveolin, and syntaxin indirectly interact with dysferlin [2,3,4,5,6,7,8]. In the present work, we wanted to ascertain whether dysferlin directly binds key proteins in the formation of a protein repair complex, as well as determine the strength of binding and its calcium dependency. Dysferlin is a major representative in a family of genes coding for proteins called ferlins, of which there are six in vertebrates. The ferlin family is characterized by C2 domains, including those associated with calcium-dependent membrane fusion and repair. The C2 domains are calcium-binding motifs of approximately 130 residues, of wide structural and functional diversity, originally identified in the calcium-dependent isoforms of protein kinase C [9]. Dysferlin, abundantly expressed in skeletal muscle, has seven C2 domains and is approximately 240 kDa in size, with a C-terminal transmembrane domain. Dysferlin is localized at the skeletal muscle t-tubule and associates with the dihydropyridine receptor L-type calcium channel. Dysferlin is mutated in patients afflicted with Miyoshi myopathy and distal anterior compartment myopathy, as well as in LGMD2B. These mutations result in either reduction or absence of expression of dysferlin in affected individuals and consequent reduction in dysferlin protein–protein interactions with membrane repair proteins. In the present work, we examined the specific domains of dysferlin that directly bind individual proteins. The hypothesis was that [Ca^2+^] rises during injury, and the [Ca^2+^]-dependency of direct interactions predicts the molecular contribution of these interactions to membrane repair, information not afforded by co-immunoprecipitation of the protein complex (used in many previous studies) which additionally includes indirect interactions. Our goal was eventually to enable enhancement of identified, critical repair complex components dependent on [Ca^2+^] elevation, serving as a measure of the repair processes. On the basis of conserved ferlin homology between otoferlin [10] and dysferlin, we also identified new dysferlin-interacting proteins, PDCD6 and FKBP8, which are known to participate in apoptotic and anti-apoptotic processes [11,12,13]. Direct interaction between individual proteins was quantitatively studied by surface plasmon resonance (SPR) analysis [14,15,16,17] as a function of calcium concentration, a presumptive indicator of membrane repair. SPR, a quantitative binding technique, was chosen over qualitative binding methods such as yeast two-hybrid and pull-down assays. Dysferlin-interacting proteins were immunolocalized in skeletal muscle by the avidin–biotin–complex peroxidase method (ABC) and colocalized with confocal Z-stack (0.6–1.0 µm) immunofluorescence microscopy, yielding results consistent with those obtained by SPR.

## 2. Results

### 2.1. Surface Plasmon Resonance (SPR) Analysis of Dysferlin Direct Protein–Protein Interactions Implicated in Dysferlin-Mediated Membrane Repair

**Figure 1:**
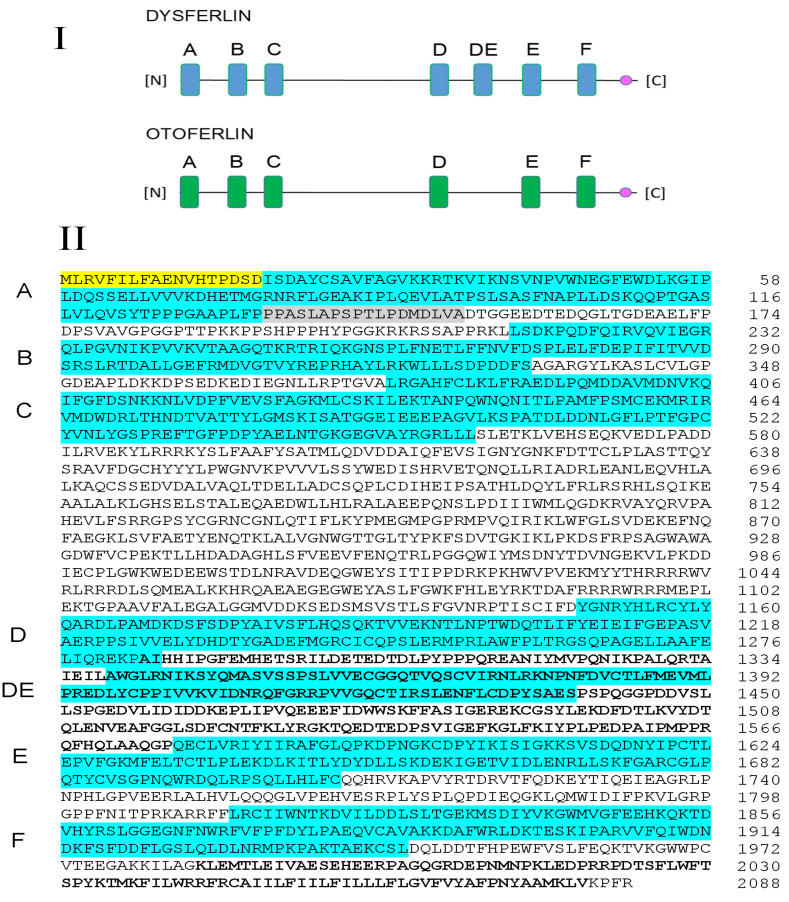
(**I**) Comparison of C2 domains (blunted rectangles) for dysferlin and the related protein, otoferlin. Ferlins are carboxy tail-anchored transmembrane proteins, with 15–20 extracellular amino acids extending, according to Kerr et al. [21], into the t-tubule lumen, i.e., in an extracellular milieu, past a predicted transmembrane helix (circle). Dysferlin and otoferlin are the best-characterized of the ferlins and reflect typical ferlin structure. Dysferlin contains a seventh C2 domain, DE, not present in otoferlin. The C2 domains are labeled according to the convention of Lek et al. [18], a convention which was followed in the present study. (**II**) Complete dysferlin sequence based on Rattus norvegicus dysferlin isoform 19: XP_006236835.1 (nucleotides: XM_006236773.4). Dysferlin C2 protein constructs, highlighted in blue, correspond to the C2 domains synthesized in this investigation including canonical cC2A and C2F domains used in binding studies. The first 124 amino acids are considered to be canonical cC2A sequence [22]. According to X-ray crystallographic analysis by Fuson et al. [22], the canonical version of dysferlin C2A has a single high-affinity Ca^2+^ binding site (discussed in [23]) and overall would coordinate two divalent cations (Ca^2+^) via amino acid residues Asp-18, Ile-19, Asp-21, Asn-40, Asp-71, His-72, and Glu-73. Beta-strands β2-β8 are included in cC2A sequence highlighted in blue. As described in the text, the uppermost yellow, blue, and grey regions (plus 24 additional aa, not illustrated) = “construct 1”; yellow and blue regions = “construct 2”; blue region alone = “construct 3”. The first bolded region (below) was used for dysferlin/PDCD6 binding investigations. The second bolded region was used for dysferlin/FKBP8 investigations.

The C2 domains in dysferlin are diagrammatically compared to those of the related ferlin, otoferlin, in Figure 1I. In the present report, we followed the C2 nomenclature described by Lek et al. [18] for dysferlin, C2A, B, C, D, DE, E, and F, based on domain similarity for otoferlin, C2A, B, C, D, E, and F. The amino acid sequences encompassing the seven dysferlin C2 domains (blue shading, labeled) within a complete dysferlin amino acid sequence are presented (Figure 1II); C2A and C2F were chosen to represent the most essential binding domains of the dysferlin molecule [19,20]. Three versions of canonical C2A were utilized in the present investigation (from dysferlin XP_006236835.1; corresponding nucleotides: XM_006236773.4), indicated by (1) the uppermost amino acid sequence highlighted in blue, referred to as construct 3; (2) extended sequence including the amino terminus highlighted in yellow plus the sequence highlighted in blue (aa 1 through aa 134), referred to as construct 2; and (3) extended sequence including the yellow, blue, and grey highlighted regions (aa 1 through 152), plus 24 aa (not illustrated) found in Rattus rattus dysferlin, transcript variant X7, XP_032762110, overall referred to as construct 1. Bolded regions in Figure 1 were used for PDCD6 and FKBP8 binding studies, respectively. The first bolded region (aa 1284–1576), which includes the entire blue C2DE region (aa 1339–1438), was utilized for dysferlin/PDCD6 binding investigations. The second bolded region at the carboxy terminus (aa 1985–2077) was utilized for dysferlin/FKBP8 investigations. Primers for PCR and constructs for binding proteins are found in Tables S1 and S2.

**Figure 2:**
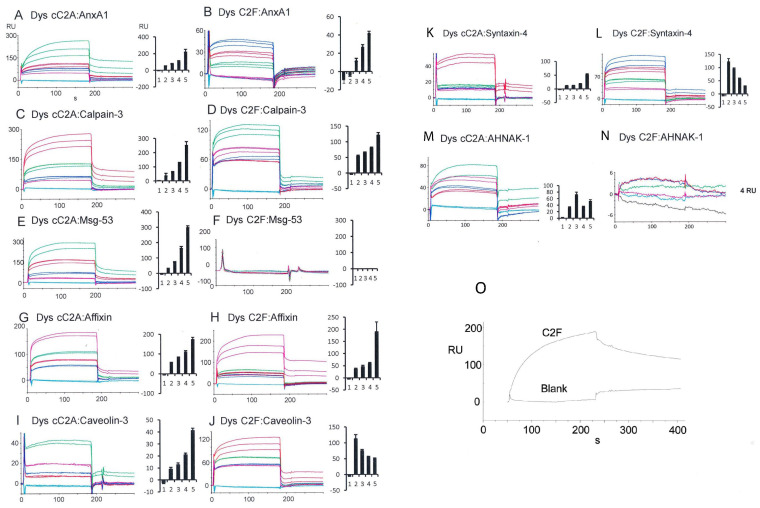
SPR analysis of dysferlin C2A (canonical) and C2F interactions with putative repair proteins. Given that Ca^2+^ concentration rises during injury, the Ca^2+^ dependency of these interactions (bar graphs) would predict the contribution of the interactions to membrane repair. SEM and n are presented in Table 1. Annexin-A1, calpain-3, mitsugumin-53, affixin, and caveolin-3 interactions were positively correlated with Ca^2+^ concentration, primarily targeting canonical C2A (cC2A), required for membrane repair. Negative Ca^2+^ dependency was observed for dysferlin C2F in interacting with caveolin-3 and syntaxin-4, suggesting a contribution at low elevations of Ca^2+^, perhaps in initial steps of repair, but a lesser contribution to membrane repair with a massive elevation of calcium. Apparently C2F does not participate in interactions with mitsugumin-53 or AHNAK1. The order of bars 1-5 for each set of proteins is: 1 = HBS-N; 2 = 1 mM EGTA; 3 = 20 µM Ca^2+^; 4 = 50 µM Ca^2+^; 5 = 100 µM Ca^2+^. (**A**) Dys cC2A: AnxA1: green = 100 µM Ca^2+^; red = 50 µM Ca^2+^; blue = 20 µM Ca^2+^; magenta = 1 mM EGTA; cyan = HBS-N. (**B**) Dys C2F: AnxA1: blue = 100 µM Ca^2+^; red = 50 µM Ca^2+^; green = 20 µM Ca^2+^; magenta = 1 mM EGTA; cyan = HBS-N. (**C**) Dys cC2A: Calpain-3: red = 100 µM Ca^2+^; green = 50 µM Ca^2+^; blue = 20 µM Ca^2+^; magenta = 1 mM EGTA; cyan = HBS-N. (**D**) Dys C2F: Calpain- 3: green = 100 µM Ca^2+^; magenta = 50 µM Ca^2+^; blue = 20 µM Ca^2+^; red = 1 mM EGTA; cyan = HBS-N. (**E**) Dys cC2A: Mitsgumin-53: green = 100 µM Ca^2+^; red = 50 µM Ca^2+^; blue = 20 µM Ca^2+^; magenta = 1 mM EGTA; cyan = HBS-N. (**F**) Dys C2F: Mitsgumin-53: grey = 100 µM Ca^2+^; red = 50 µM Ca^2+^; cyan = 20 µM Ca^2+^; magenta = 1 mM EGTA; green = HBS-N + 1 mM EGTA; blue = HBS-N. (**G**) Dys cC2A: Affixin: magenta = 100 µM Ca^2+^; green = 50 µM Ca^2+^; red = 20 µM Ca^2+^; blue = 1 mM EGTA; cyan = HBS-N. (**H**) Dys C2F: Affixin: magenta = 100 µM Ca^2+^; green = 50 µM Ca^2+^; red = 20 µM Ca^2+^; blue = 1 mM EGTA; cyan = HBS-N. (**I**) Dys cC2A: Caveolin-3: green = 100 µM Ca^2+^; magenta = 50 µM Ca^2+^; blue = 20 µM Ca^2+^; red = 1 mM EGTA; cyan = HBS-N. (**J**) Dys C2F: Caveolin-3: red = 1 mM EGTA; green = 20 µM Ca^2+^; blue = 50 µM Ca^2+^; magenta = 100 µM Ca^2+^; cyan = HBS-N. (**K**) Dys cC2A: Syntaxin-4: red = 100 µM Ca^2+^; green = 50 µM Ca^2+^; blue = 20 µM Ca^2+^; magenta = 1 mM EGTA; cyan = HBS-N. (**L**) Dys C2F: Syntaxin-4: blue = 1 mM EGTA; red = 20 µM Ca^2+^; green = 50 µM Ca^2+^; magenta = 100 µM Ca^2+^; cyan = HBS-N. (**M**) Dys cC2A: AHNAK1:green = 20 µM Ca^2+^; magenta = 1 mM EGTA; blue = 100 µM Ca^2+^; red = 50 µM Ca^2+^; cyan = HBS-N. (**N**) Dys C2F: AHNAK1: blue = 1 mM EGTA; red = 100 µM Ca^2+^; green = 50 µM Ca^2+^; magenta = 20 µM Ca^2+^; cyan = 1 mM EGTA; grey = HBS-N. Binding partners are presented as ligand:analyte. Analyte concentration in each case was 100 nM. (**O**) SPR analysis of dysferlin C2F compared to BSA blank (upper curve, mobile C2F ferlin domain with immobilized syntaxin-1 construct vs. lower curve, mobile BSA with immobilized syntaxin-1) demonstrating interaction specificity.

SPR analysis of direct protein–protein interactions for dysferlin cC2A and C2F domains with specific motifs of individual putative repair proteins is presented in Figure 2 and summarized in Table 1. Equilibrium/steady-state binding to dysferlin was found to be strongest, overall, for the proteins annexin A1, calpain-3, mitsugumin-53, and caveolin-3 and in the range of 10^−7^–10^−9^ M *K_D_*. Affixin and syntaxin-4 showed comparatively weaker binding (10^−5^–10^−7^ M *K_D_*). Differential interaction between cC2A and C2F was most obvious for the large protein constructs such as mitsugumin-53 and AHNAK1, which only exhibited binding to dysferlin cC2A and not C2F. In contrast, calpain-3 in particular, exhibited strong binding to dysferlin C2F.

Protein–protein interactions displayed differential dependency on [Ca^2+^]. The presumptive repair proteins, whose interactions with dysferlin were positively correlated with [Ca^2+^] as predicted during repair, included annexin A1, calpain-3, mitsugumin-53, affixin, and caveolin-3. These interactions primarily recognized dysferlin cC2A, whereas negative [Ca^2+^] dependency was observed for dysferlin C2F in interacting with caveolin-3 and syntaxin-4 (Figure 2 and Table 1). The dysferlin C2F interactions with caveolin-3 and syntaxin-4 may therefore reflect conditions existing at basal/slightly elevated calcium levels during resting conditions. Bovine serum albumin served as a negative control, not interacting with dysferlin C2F in SPR (Figure 2O).

For K_D_ values (dissociation constants), nd = not detectable. SEM = standard error of the mean. Calcium dependence of binding of the protein partners (rightmost column) is indicated by 0 (≤ 7% between 0 and 100 μM Ca^2+^, the highest calcium concentration studied), + (26–55% between 0 and 100 μM Ca^2+^), ++ (78–110% between 0 and 100 μM Ca^2+^), or +++ (184–247% between 0 and 100 μM Ca^2+^), or − (negative calcium dependence).

### 2.2. Dysferlin Interacts with Proteins Related to Apoptosis/Anti-Apoptosis, Linking PDCD6 and FKBP8 Pathways

**Figure 3:**
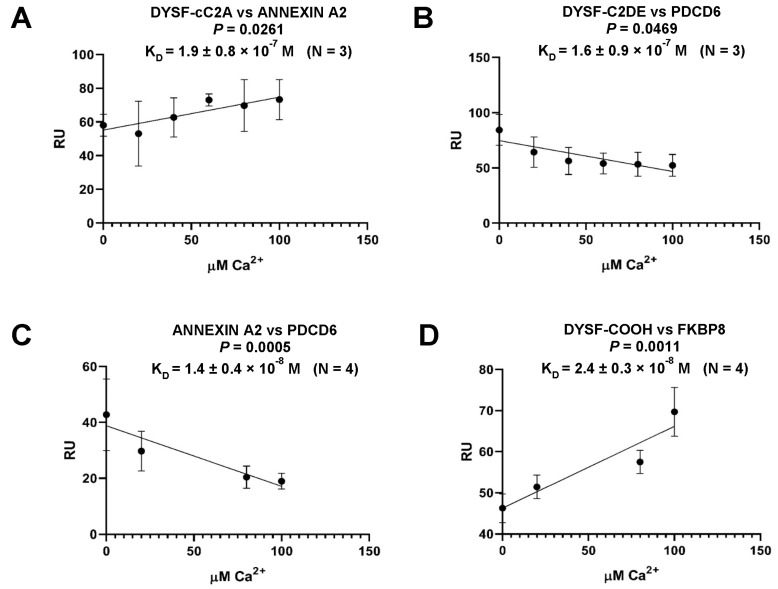
Calcium-dependent protein–protein interactions of dysferlin related to apoptotic and anti-apoptotic pathways. SPR response units (RU) are plotted vs. Ca^2+^ concentration for analyte concentration of 100 nM. Error bars refer to the standard deviation of the mean for the response unit (RU) at each Ca^2+^ concentration. Results were statistically analyzed by linear regression software (Graph Pad Prism, San Diego, CA). Probability, P, by linear regression analysis, indicates the degree to which the slope of each line significantly differs from zero. Standard errors for the slopes were (**A**) 0.080, (**B**) 0.130, (**C**) 0.048, and (**D**) 0.049. Each *K_D_* value (0 added Ca^2+^) is presented as mean ± standard error of the mean. (**A**,**D**) show positive calcium dependence, whereas (**B**,**C**) show negative calcium dependence. (**B**) PDCD6, associated with apoptotic pathways, interacts with dysferlin by SPR. (**C**) Annexin-A2, known to function as a membrane organizer, may shed bound apoptotic PDCD6 as calcium increases with muscle injury. (**D**) Anti-apoptotic FKBP8 interacts with dysferlin, as measured by SPR. The FKBP8 response may be favored with calcium increase in muscle injury. DYSF-cC2A, DYSF-C2DE, and DYSF-COOH constructs are defined in the results. Stated calcium levels are similar to free calcium levels, since the low protein concentrations employed (nM levels) would not alter calcium concentrations (by protein binding) to any significant extent (cf. [26]).

As a member of the ferlin superfamily with known homology to otoferlin (Figure 1), dysferlin was examined for otoferlin-like protein–protein interactions that had previously been identified by yeast two-hybrid analysis in which PDCD6 and FKBP8 were recognized as otoferlin binding partners [24]. Blast comparison confirmed the specific motif similarities between otoferlin and dysferlin. We produced a construct for the extended dysferlin C2DE region (Figure 1, amino acids 1284-1577) based upon amino acid alignment for otoferlin and dysferlin. We found dysferlin C2DE interacted with PDCD6 (Figure 3B), and the interaction was characterized as having a minimal negative calcium dependence. Similarly, we produced a dysferlin construct for amino acids 1985–2077 (Figure 1, carboxy terminal region) which was utilized for dysferlin/FKBP8 investigations (Figure 3D). The carboxy terminus of dysferlin interacted with the carboxy terminus of anti-apoptotic FKBP8, but in contrast with PDCD6/dysferlin, the FKBP8/dysferlin interaction displayed strong positive calcium dependence (Figure 3D).

Annexin A2, a recognized dysferlin binding protein, was also investigated given the reported interaction of annexins A7 and A11 with PDCD6 [25], the conservation of amino termini across annexins including annexin A2, and therefore the likely interaction of annexin A2 with PDCD6. Annexin A2, in fact, strongly interacted with PDCD6 (Figure 3C) and with negative Ca^2+^ dependence. Annexin A2 also directly interacted with dysferlin, however in this case with positive Ca^2+^ dependence (Figure 3A). The results would be consistent with a calcium-induced switch in alternate protein complexes accompanying a molecular response to skeletal muscle injury reflecting a presumptive injury path to cell destruction (apoptosis) and membrane recovery (anti-apoptosis), linked to dysferlin. At low/basal Ca^2+^ levels, dysferlin would form a complex with PDCD6 and annexin A2, the latter two strongly binding to each other. With elevation of Ca^2+^, as occurs in injury, dysferlin would dissociate from PDCD6 (Figure 3B) and form a strong interaction with FKBP8 (Figure 3D). Annexin A2 would be released from PDCD6, with annexin A2 available to interact with dysferlin (Figure 3A) and PDCD6 available to associate with its PDCD6-interacting protein (PDCD6IP).

### 2.3. Calcium-Binding Properties of Extended cC2A Construct

**Figure 4:**
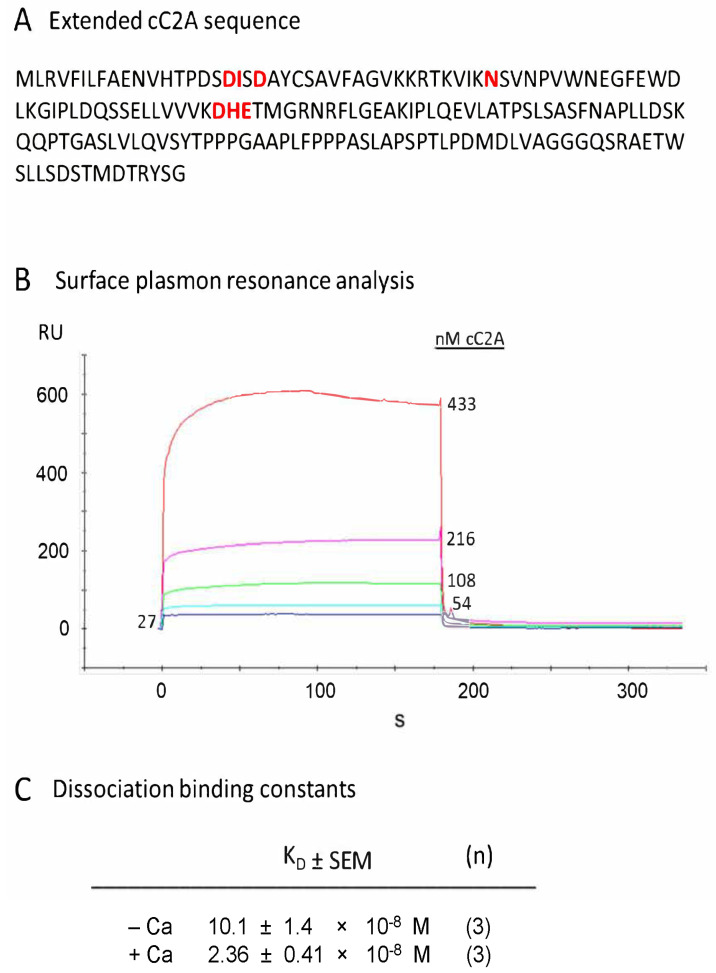
Dysferlin canonical C2A construct 1 containing main presumptive calcium-binding residues: demonstration of calcium dependence by SPR. (**A**) Amino acid sequence of construct 1 (cf. Figure 1). Residues thought essential for calcium binding are shown in red letters. (**B**) Representative example of SPR plot (0 added calcium). Plot shows annexin A2 (ligand) interaction with cC2A (analyte) for varying levels of analyte. (**C**) *K_D_* values for cC2A–annnexin–A2 interaction in the presence and absence of 100 µM calcium. Note lower *K_D_* value (tighter binding) with calcium, indicating calcium dependence (unpaired two-tailed *t*-test, *p* = 0.0064). Similar binding results were obtained when analyte and ligand were reversed.

The canonical C2A region of dysferlin was synthesized to include the yellow, blue, and grey regions of Figure 1 (as described in the Figure 1 legend and Figure 4A) to test whether the inclusion of aa 1-18 (containing Asp-18) with the full complement of other putative C2A calcium-binding residues (see Discussion; [22,23]) would significantly alter the calcium dependence for cC2A binding with other proteins (Table 1), here with annexin A2 used as a binding partner. We demonstrated that construct 1 (Figure 4A,C) exhibited calcium dependence, as was observed for the shorter cC2A construct 3, which lacked Asp-18 (Figure 1 and Figure 3A for comparison of amino acids). Consistency of results for extended (construct 1) versus the shorter cC2A (construct 3) was further emphasized by similar RU and *K_D_* values for both Figure 4B (at 108 nM cC2A) and Figure 3A (100 nM cC2A; no added Ca^2+^). Ca^2+^ dependency by linear regression analysis of the shorter version was paralleled by Ca^2+^ dependency of *K_D_* values demonstrated for the extended version, *p* = 0.0064 (highly statistically significant).

### 2.4. Localization of Putative Dysferlin Repair-Linked Proteins in Myofibers

**Figure 5:**
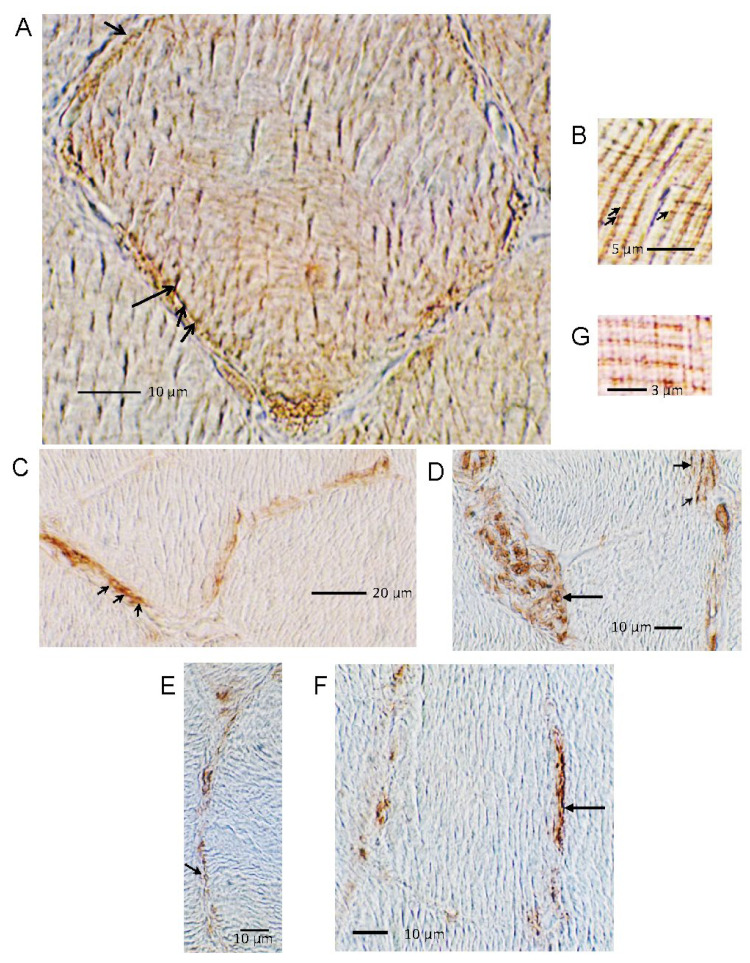
Dysferlin and annexin A2 detected with 3,3′-diaminobenzidine (DAB) chromogen staining in skeletal muscle sarcolemma and regions adjacent to Z-disks. (**A**) Magnified transverse section of muscle fiber showing dysferlin enriched at or near the sarcolemma. Dysferlin immunoreactivity extends intracellularly from the sarcolemma via the t-tubule system (long arrow) and also is present as punctate immunostaining on mitochondria adjacent to the sarcolemma (short arrows). (**B**) Longitudinal section of muscle fiber, diagonally aligned, showing repeating bands of sarcomeres and dysferlin staining to either side of sarcomere Z-disks (small black arrows point to doublet of immunoreactivity as reported by others [27]). (**C**) Annexin A2 immunoreactivity on sarcolemma and surrounding adjacent mitochondria (short arrows). (**D**) Layered staining at sarcolemmal surface of myofibers consistent with annexin A2 associated with region of invagination of t-tubules (short arrows). Nerve end plate with heavy expression of annexin A2 in presumptive mitochondria (long arrow). (**E**) Transverse section with annexin A2 immunoreactivity on the sarcolemma and (**F**) associated with t-tubule system (arrow). (**G**) Longitudinal section with annexin A2 immunoreactivity adjacent to Z-line. Annexin A2 immunolocalizations were carried out with SC-1924 antibody (Santa Cruz Biotechnology). The “Hamlet” antibody for dysferlin was procured from Leica Biosystems (Wetzlar, Germany).

Dysferlin was immunolocalized with 3,3′-diaminobenzidine chromogen and the avidin biotin complex (ABC method, Vector Labs, Newark, CA, USA), enabling detection of intracellular morphology. Immunoreactivity was observed on the sarcolemma in transverse sections of the myofibers, extending intracellularly in the t-tubule system (Figure 5A). Punctate immunoreactivity was observed on the sarcolemma in close proximity to mitochondria (Figure 5A). The myofiber nuclei were not immunoreactive for conditions reflecting basal Ca^2+^. In longitudinal sections, we reproduced localization of dysferlin to two rows of punctate depositions on either side of the Z-line (Figure 5B), as reported by Roche et al. [27]. Annexin A2 with diaminobenzidine (DAB) (Figure 5C–G) was localized to the sarcolemma and appeared to encompass myofiber mitochondria close to the sarcolemma (Figure 5C). Annexin A2 was strongly expressed in nerve fibers at the neuromuscular junction end plates and was associated, in particular, with the mitochondria (Figure 5D). Annexin A2 appeared to immunolocalize to sites on either side of the Z-line with DAB detection (Figure 5G).

**Figure 6:**
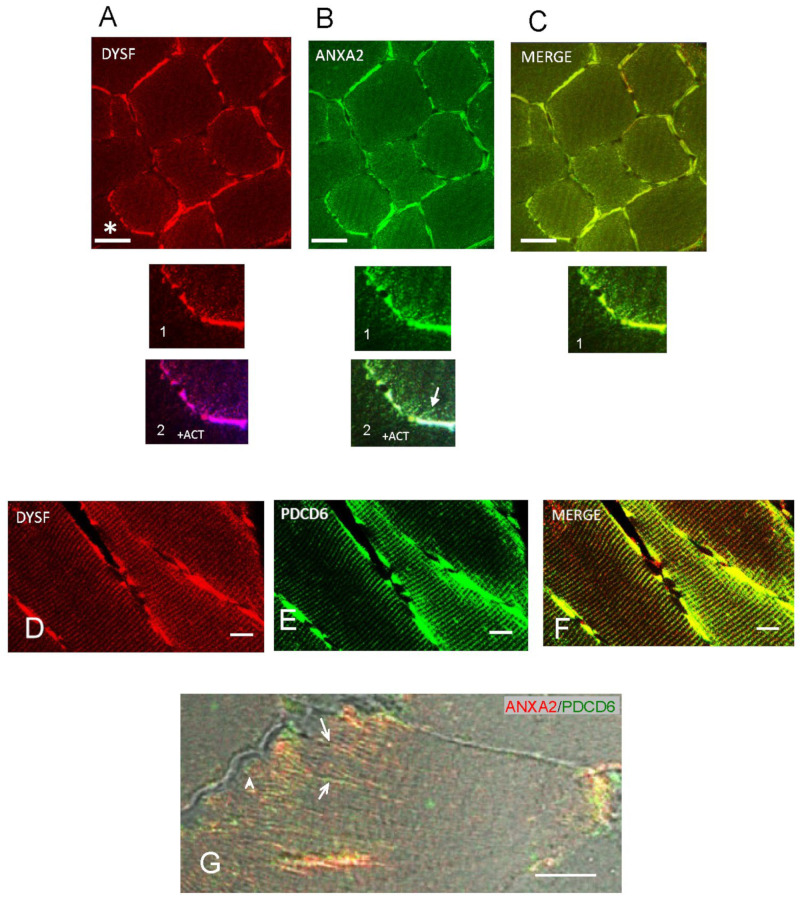
Dysferlin, annexin A2, and PDCD6 immunofluorescence labeling of myofibers. (**A**) Dysferlin immunoreactivity (red), showing prominent localization in the sarcolemma in transverse sections. (**B**) Annexin A2 immunoreactivity (green) (SC-1924 antibody, Santa Cruz Biotechnology), showing localization in the sarcolemma and in sarcoplasmic reticulum (enhanced in some cells). (**C**) Merging of immunoreactivity of dysferlin and annexin A2. Scale bar = 20 µm for (**A**–**C**). (**A1**) Enlarged image of A* region, showing dysferlin and (**A2**) dysferlin colocalized with actin in t-tubules extending from the sarcoplasm. (**B1**) Enlarged image of annexin A2 and (**B2**) annexin A2 colocalized with dysferlin and actin in t-tubules (arrow). (**C1**) Magnified view of dysferlin and annexin A2 merged in t-tubules. (**D**–**F**) Dysferlin and PDCD6 immunofluorescence in Z-stack confocal microscopy of longitudinal sections of skeletal muscle. (**D**) Dysferlin immunoreactivity (red), consistent with localization on either side of Z-disks (cf. [27]). (**E**) PDCD6 immunoreactivity (green). (**F**) Dysferlin and PDCD6 merged, showing (particularly when viewed at high digital magnification) overlapping red and green immunofluorescence on the sarcolemma. Scale bar = 5 µm for D-F. (**G**) Colocalization of annexin A2 (red) (annexin A2 mouse monoclonal antibody 66035-1-Ig, Clone No. 1C1E12, Proteintech) and PDCD6 (green) on t-tubules (arrows) in brightfield immunofluorescence image of transverse section. Note arrangement of PDCD6 (green) (arrowhead) and associated annexin A2 (red) in region of subsarcolemmal mitochondria. Scale bar = 5 µm.

Confocal Z-stack immunofluorescence microscopy indicated colocalization of dysferlin with annexin A2 and actin (see Figure 6 legend) on the sarcolemma membrane but not within the nuclei in transverse sections (Figure 6A,B,B2), again for control/basal levels of Ca^2+^. The t-tubule system emanating from the sarcolemma membrane sites indicated colocalization of dysferlin with actin (Figure 6A2) and annexin A2 with actin (Figure 6B2), with overlapping immunoreactivity of dysferlin and annexin A2 (Figure 6C1).

PDCD6, the apoptosis-linked gene-2 (ALG-2) protein which interacts with dysferlin (at basal Ca^2+^ levels, Figure 3B), was immunolocalized with Z-stack confocal fluorescence microscopy to sarcolemma membrane sites in longitudinal myofiber sections (Figure 6E), with immunofluorescence overlapping that of dysferlin (Figure 6D; merged, Figure 6F). Annexin A2 and PDCD6 appeared to colocalize in the t-tubule system (Figure 6G).

**Figure 7:**
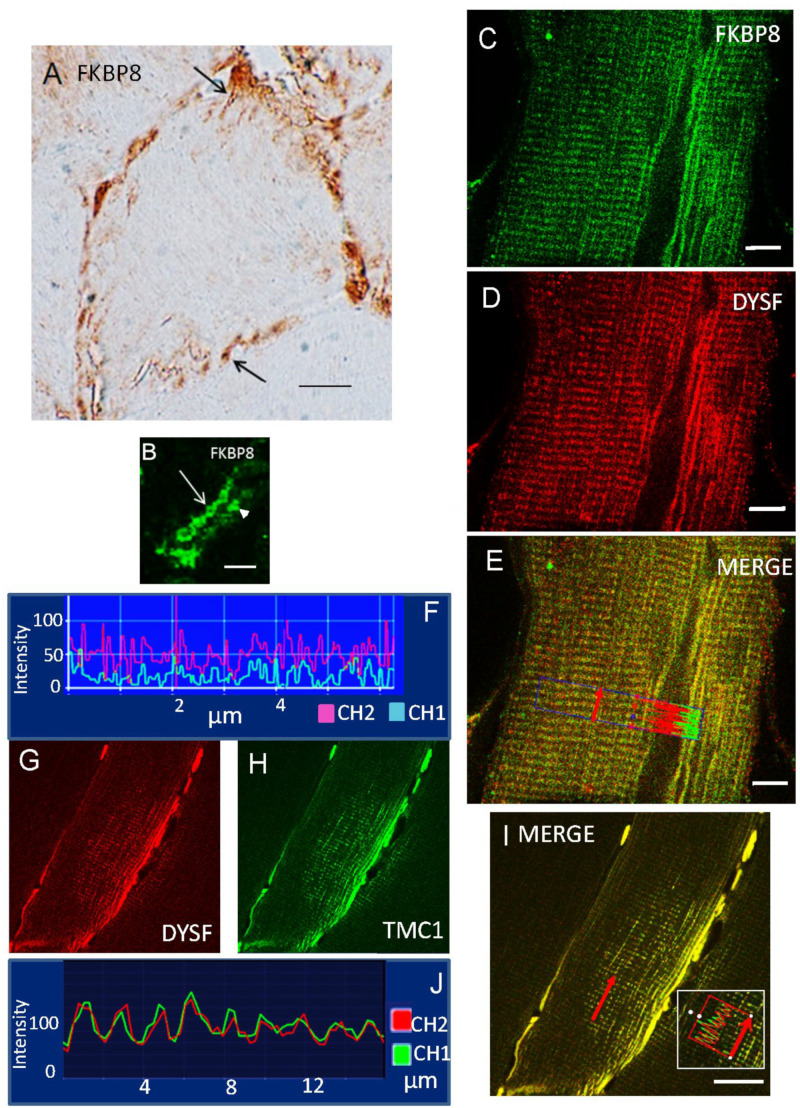
FKBP8 (FKBP38) localization compared with that of dysferlin in skeletal muscle. (**A**) FKBP8, as a marker protein for the outer membrane of mitochondria, was identified in a transverse section of a myofiber in rat skeletal muscle, detected with 3,3′-diaminobenzidine. FKBP8 immunoreactivity was found in direct association with the sarcolemma extending intracellularly (arrows), a presumptive member of the subsarcolemmal population of mitochondria [28]. Scale bar = 10 µm. (**B**) Magnified view of FKBP8 immunofluorescence surrounding mitochondria, identified by size, in close apposition to the sarcolemma (arrow). The arrowhead identifies the sarcolemma of adjacent cell. Scale bar = 5 µm. (**C**) FKBP8 (green), longitudinal section. (**D**) Dysferlin (red), longitudinal section. (**E**) FKBP8 and dysferlin colocalized (merged = yellow) in longitudinal section (**C**–**E**, scale bars = 6 µm). (**E**,**F**) Computer sampling of fluorescence intensity across rows of sarcomere Z-discs delineated in E (red arrow) indicates spatial fluorescence coincidence for FKBP8 (CH1, blue-green) and dysferlin (CH2, pink-red) (**F**). (**G**–**J**) Z-stack confocal immunofluorescence for TMC1 (**H**), a putative mechanosensory channel subunit [29] which is reported to directly bind the carboxy terminus of FKBP8 (which would be in competition with dysferlin), compared to dysferlin (**G**) and merged (**I**) (scale bar = 16 µm.) (**J**) High spatial coincidence of fluorescence intensity was observed across rows of sarcomere Z-disks (red arrow; inset in I) for TMC1 (CH1) and dysferlin (CH2). FKBP8 immunolocalizations were carried out with ab24450 antibody (ABCAM, Cambridge, MA, USA).

FKBP8, whose interaction with dysferlin was strongly enhanced with an increase in Ca^2+^ concentration (Figure 3D), is a well-known outer membrane mitochondrial marker, also observed for the subsarcolemmal population of mitochondria in the present investigation with DAB (Figure 7A) and immunofluorescence (Figure 7B). Extension of skeletal muscle mitochondria from the sarcolemma membrane intracellularly has been noted (Figure 7A, arrows), consonant with description of “elongated tubules” [28]. Confocal immunofluorescence Z-scan longitudinal images (1 µm slice depth) indicated that FKBP8 (Figure 7C) partially colocalized with dysferlin (Figure 7D–F) at sites on either side of the Z-line. As predicted by SPR with direct binding of FKBP8 and PDCD6 to dysferlin, FKBP8 compartmentalized with PDCD6 in myofibers by immunohistochemistry. FKBP8, in addition, is known to interact directly with TMC1, a mechanosensory ion channel candidate [29]. In the present investigation, TMC1 was found to colocalize closely with dysferlin in longitudinal sections (Figure 7G–J).

### 2.5. Dysferlin C2 Domain Interactions and Dependence on [Ca^2+^]

**Figure 8:**
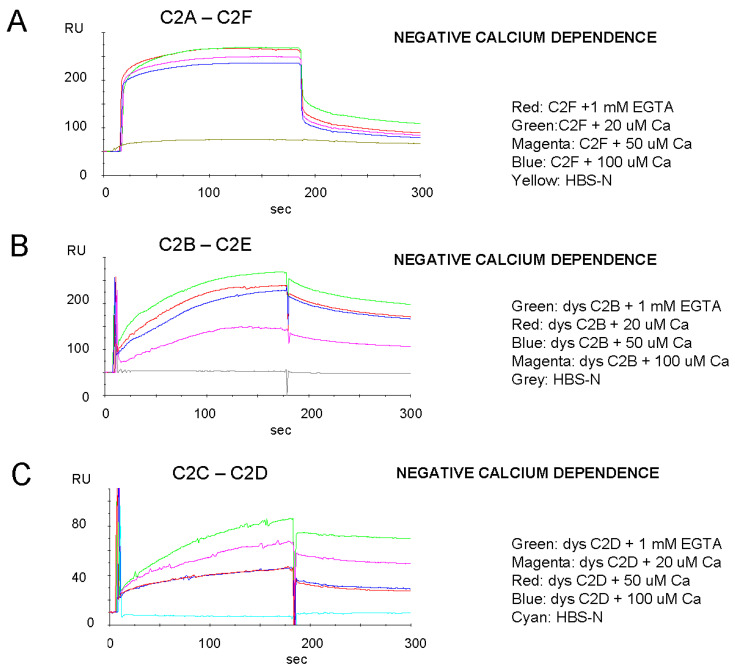
Dysferlin C2 domains bind other dysferlin C2 domains (heterodomain binding) and show negative calcium dependence. Pairs of representative dysferlin domains were examined via SPR as shown. (**A**–**C**) All three heterodomain interactions examined indicated negative calcium dependence, suggesting configuration changes of the native dysferlin molecule with changes in calcium level (see Conclusions).

Dysferlin C2-C2 domain interactions were investigated involving pairing of representative C2 domains (Figure 8) based on previous determinations of self-binding for the homologous ferlin, otoferlin, which would contribute to Ca^2+^-dependent function [10]. By SPR, dysferlin cC2A was found to bind dysferlin C2F, C2B bound C2E, and C2C bound C2D, all with negative calcium dependence, i.e., the binding strength increased as the calcium concentration was lowered (Figure 8A–C), compatible with a model of injury-induced expansion of dysferlin molecules as the Ca^2+^ concentration is raised.

## 3. Discussion

### 3.1. Binding of Dysferlin C2 Domains to Repair Complex Proteins

In this study, we examined the interactome of dysferlin C2 domains by designing and synthesizing fusion proteins of dysferlin and domains from other proteins, known from previous reports to interact with dysferlin. We quantitatively ascertained direct binding of protein partners at different calcium concentrations by SPR analysis. We also tested the interaction of dysferlin with the pro-apoptotic protein, PDCD6, and the anti-apoptotic protein, FKBP8, based on the interaction of these proteins reported for the related ferlin, otoferlin [24], and the homologous amino acid sequences existing in the two ferlins. Immunofluorescence colocalization of binding partners in confocal Z-stack 1 µm slices of rat skeletal muscle was examined for consistency with SPR findings. We finally proposed a model of membrane repair after injury, based on our current findings and those of prior studies.

SPR analyses of direct interaction of dysferlin C2A (canonical) and C2F with putative repair proteins were compared (Figure 2). We chose the canonical C2A (cC2A) [22], due to its predicted calcium and phospholipid-binding properties, whereas the non-canonical isoform is predicted to be calcium-independent under physiological conditions. The canonical version of dysferlin C2A has a single high-affinity Ca^2+^ binding site [23]. C2F interactions were examined in accord with the recognized role of C2F in the homologous ferlin, otoferlin, in coupling to vesicular components [30].

Overall, we observed calcium dependency for interactions of dysferlin C2 domains with repair complex proteins. Annexin A1, calpain-3, mitsugumin-53, affixin, and caveolin-3 interactions with dysferlin cC2A (aa 19-134, Figure 1) were positively correlated with Ca^2+^ concentration required for membrane repair (Figure 2 and Table 1). On the other hand, negative calcium dependency was observed for dysferlin C2F in interacting with caveolin-3 and syntaxin-4, suggesting a contribution at physiologically low elevations of calcium, perhaps in initial steps of repair, but a lesser contribution to membrane repair and protein unfolding with a massive elevation of calcium in injury.

### 3.2. Dysferlin-Binding Proteins Whose Ca^2+^-Dependent Binding Would Influence Cell Membrane Repair

Annexins are calcium- and phospholipid-sensitive proteins which can function as organizers of membrane domains [31,32,33,34], shown to enhance plasma membrane resealing in diverse cultured cell types [35,36,37] with isoform-specific function and trafficking [3,38,39]. Annexin-A1 showed positive calcium dependence for binding the dysferlin cC2A and C2F domains (Table 1). Co-immunoprecipitation has revealed that annexin-A1 associates with dysferlin in a calcium- and membrane-injury-dependent manner [2], a behavior consistent with reported involvement of multiple annexins in muscle repair [35] and muscular dystrophy (e.g., annexin A11).

Calpain3 (CAPN3) [40], a protease hypothesized to cleave dysferlin partners annexin A1 and annexin A2 [2], directly interacted with dysferlin cC2A and C2F, both positively dependent on calcium (Table 1). The CAPN3 interaction with dysferlin C2F in particular (and unusually for dysferlin C2F interactions) showed high affinity (*K_D_* = 2.2 ± 0.6 × 10^−9^ M). Based upon molecular similarity to otoferlin, this interaction may relate to regulation of vesicle release [30].

Mitsugumin-53 binds the cC2A region of dysferlin (Table 1). In interacting with dysferlin, mitsugumin-53 constitutes an essential part of acute membrane repair machinery [41], facilitating vesicle translocation to the sites of membrane injury.

Affixin (beta-parvin) is an integrin-linked kinase-binding protein that is involved in the linkage between integrin and the cytoskeleton. Our SPR findings (Figure 2 and Table 1) and previously published interaction data (4) suggest that affixin may act with dysferlin in membrane repair as part of a dysferlin complex. Affixin colocalizes with dysferlin at the sarcolemma of normal human skeletal muscle [4]. The N-terminal calponin-homology domain of affixin is a binding site for dysferlin and the C-terminal region of dysferlin has an apparent affixin binding site, as evidenced by previous immunoprecipitation studies with deletion mutants (4), consistent with positively Ca^2+^-dependent dysferlin–C2F interaction we observed (Figure 2H).

Caveolin-3 is a skeletal muscle membrane protein which is important for the formation of caveolae. With SPR, we found that caveolin-3 interacted with dysferlin cC2A with positive calcium dependence and C2F with negative calcium dependence, suggesting a calcium-dependent structural rearrangement of dysferlin with caveolin-3 (Figure 2 and Table 1). Dysferlin is known to co-immunoprecipitate with caveolin-3 in isolates of biopsied human skeletal muscle, suggesting a functional interaction [42].

Syntaxin-4, another repair protein, is predominantly localized to the plasma membrane in both skeletal muscle and myotubes in culture [43] and is implicated in the delivery of trans-Golgi network cargo to the cell surface. In our investigation, dysferlin interacted with syntaxin 4 (Table 1), however with differential dependence on calcium (Figure 2). The interaction of cC2A was positively calcium-dependent, implying a role in membrane repair with elevation of calcium, whereas dysferlin C2F binding was negatively impacted by calcium concentration, with contribution at primarily low elevations of calcium. On the basis of our previously identified association between otoferlin and syntaxin-1 in cochlear inner hair cells [44], Evesson et al. [45] suggested that syntaxin-4 serves as a receptor protein for intracellular vesicles targeted to the muscle plasma membrane consistent with co-immunoprecipitation of syntaxin-4 and dysferlin [46].

AHNAK1, a large (700 kDa) phospho-nucleoprotein (also called desmoyokin) is thought to be a key player in interactions with dysferlin and myoferlin. In the present work (Table 1 and Figure 2), we ascertained an interaction between dysferlin cC2A and the final 500 amino acids of AHNAK1, in agreement with specificity previously reported [47]. The K_D_ was 3.8 ± 1.6 × 10^−7^ M for AHNAK1 interaction with dysferlin cC2A. Dysferlin C2F did not appear appreciably to bind AHNAK1. AHNAK is involved in cell membrane differentiation, repair, and signal transduction and forms a complex with dysferlin in skeletal muscle and in coronary arterial endothelial cells [48].

### 3.3. Dysferlin-Interacting Proteins Related to Apoptosis/Anti-Apoptosis: PDCD6 (ALG-2), FKBP8, Annexin A2, and Links to Mitochondria

There are a number of programmed cell death mechanisms, including apoptosis and necroptosis [13]. Heidrych [24] demonstrated with yeast two-hybrid analysis that the programmed cell death protein PDCD6 (ALG-2) [11,49] interacts with a region of the related ferlin, otoferlin, between C2 domains C2D and C2E. We identified a corresponding region in the dysferlin molecule, which we termed C2DE (cf. Figure 1). We found dysferlin C2DE interacted with PDCD6 (Figure 3B). PDCD6 is recognized as participating in cell membrane repair [50], although not previously as a dysferlin-interacting protein. PDCD6 is recognized to interact with annexin A7 and annexin A11 [25] and may participate in muscle wasting. Given that the amino termini are conserved across annexins, we investigated annexin A2 as well as annexin A1, since annexin A2 is co-regulated with annexin A1 in dysferlinopathic mice [2]. Annexin A2 strongly interacted with PDCD6 (*K_D_* = 1.4 × 10^−8^ M) with negative calcium dependence (*p* = 0.0005) (Figure 3C). Annexin A2 (amino terminus) also bound to dysferlin cC2A, with moderate positive calcium dependence (Figure 3A). Our *K_D_* values in Figure 3 were measured at 26.6 µM [Ca^2+^] in the SPR buffer (which we determined by inductively coupled plasma mass spectroscopy). Influx of micromolar calcium above this level during injury in vivo could thus facilitate the formation of protein pairs or change the composition of the complex at micromolar values of calcium, as shown on the abscissas of Figure 3.

**Figure 9:**
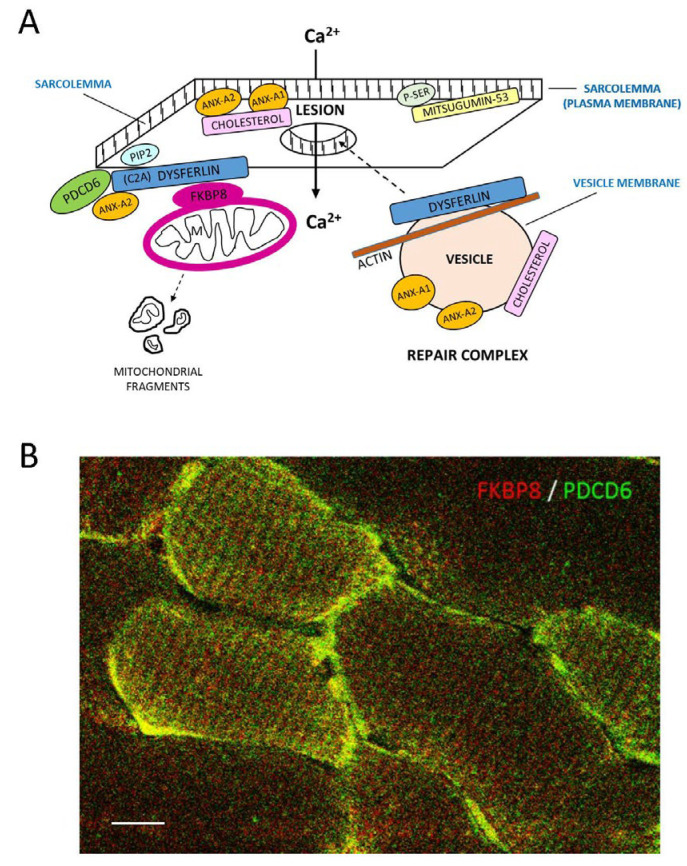
(**A**) Model of dysferlin-directed membrane repair in myofibers. Calcium enters lesion in membranes and stimulates mobilization of a dysferlin-associated repair complex that promotes membrane sealing. Dysferlin is bound to the lipidic sarcoplasmic membrane, assumedly through its cC2A domain, and also localizes to cytoplasmic vesicles. Furthermore, dysferlin associates with transverse tubules (not shown) through its cC2A domain. Dysferlin cC2A binds membrane PIP2 [56] and participates in clustering of lipids [57]. FKBP8, present in the mitochondrial outer membrane, binds the dysferlin carboxy terminal region with increasing Ca^2+^ concentration and is well positioned for muscle preservation and repair. PDCD6 binds dysferlin at a site different from that of FKBP8 and is released from its annexin-A2 partner with Ca^2+^ elevation, allowing PDCD6 to bind its interacting protein, PDCD6IP, and freeing annexin-A2 to interact strongly with dysferlin as part of a concerted repair mechanism, with mitochondrial fragmentation as a repair signal (see text). Annexins A1 and A2 bind membrane phospholipids in a calcium-dependent manner and form organizing networks on the membrane surface which recruit interacting proteins such as dysferlin [32]. Annexins also aggregate intracellular vesicles to form an endomembrane patch that is trafficked, along with dysferlin, to the site of injury [5,58]. Actin is also thought to be involved in vesicle trafficking and membrane remodeling, with calcium-induced incorporation into muscle myoblast filaments regulated by dysferlin [59]. Cholesterol is altered in the plasma membrane in response to injury and probably changes position in dysferlin-containing intracellular vesicles as well [58]. This cholesterol change is “sensed” by annexin A2, which accumulates at cholesterol-rich lipid microdomains. Mitsugumin-53 binds directly to lipid-borne phosphoserine, at the plasma membrane or in caveoli. Mitsugumin-53 is thought to help stabilize the membrane by binding and recruiting dysferlin, caveolin-3, and annexins as part of the repair process [8]. Other proteins are undoubtedly involved in repair that are not included in the diagram. (**B**) Z-scan confocal immunofluorescence indicates co-compartmentalization of FKBP8 (red) and PDCD6 (green) on the sarcolemmal membrane (yellow) at basal Ca^2+^ concentration in transverse section of skeletal muscle. Scale bar = 10 µm. FKBP8 immunolocalizations were carried out with 11173-1-AP antibody (Proteintech, Rosemont, IL, USA).

FKBP8 (FKBP38) [12,51,52] is an inherent inhibitor of calcineurin and represents an anti-apoptotic protein whose overexpression blocks apoptosis by anchoring anti-apoptotic proteins B-cell lymphoma protein (Bcl-2) and Bcl-xL to mitochondria [12]. The FKBP, or FK-binding proteins, were so named because skeletal muscle FKBP12 was shown to be the binding protein for the immunosuppressant drug FK506 [53]. FK506-binding proteins are members of the immunophilin family, which bind immunosuppressants such as rapamycin and are also part of the ryanodine receptor complex. Ca^2+^/calmodulin modulates the interaction between the FKBP8 catalytic domain and Bcl-2 [54], lowering the affinity of the FKBP8 active site for Bcl-2. FKBP8 is localized to the outer membrane of mitochondria (Figure 9A) and is also expressed in the endoplasmic reticulum. An FXXL domain in the N-terminus of FKBP8 binds to autophagosomal protein LC3A-II. With recruitment of LC3A-II, FKBP8 and Bcl-2 move from the outer mitochondrial membrane to the endoplasmic reticulum membrane via microtubule-associated vesicular transport during mitophagy (a form of autophagy responsible for the elimination of damaged mitochondria). FKBP8 and Bcl-2 thus escape the degradative fate of most mitochondrial proteins [55]. This escape of FKBP8 is dependent on the low basicity of its COOH-terminal sequence and is essential for the suppression of apoptosis during mitophagy. It has been hypothesized that a similar mechanism may be induced in muscle with stress of injury, particularly in genetically compromised muscle.

We observed a localization of FKBP8, a known marker protein for the outer mitochondrial membrane [12], on myofibers with both DAB and immunofluorescence (Figure 7B) surrounding organelles with mitochondrial-like dimensions that were consistent with those of the subsarcolemmal population of mitochondria [28] and smaller than would be predicted for nuclei.

The carboxy terminus of FKBP8 that is required for localization at the mitochondrial outer membrane sites was previously identified as interacting with the carboxy terminus of otoferlin by yeast two-hybrid analysis [24]. FKBP8 likewise strongly interacted with the carboxy terminus of dysferlin (*K_D_* = 2.4 × 10^−8^ M) (Figure 3D), consistent with homologous sequence between dysferlin and otoferlin. This interaction of FKBP8 and dysferlin was positively dependent on calcium (*p* = 0.0011) and thus would be well-positioned for muscle preservation with elevation of calcium accompanying membrane repair (Figure 9).

Mitochondria are recognized as controlling redox homeostasis, Ca^2+^ signaling, iron metabolism, innate immunity, and apoptotic cell death [60,61,62,63]. Recently, Horn et al. [64] demonstrated that mitochondria fragmentation was necessary for membrane repair initiated by Ca^2+^ influx at the injury site. The interplay between Ca^2+^ influx and mitochondrially generated reactive oxygen species (mtROS) was found to enhance actin-mediated wound closure for survival of injured mammalian muscle and non-muscle cells. Calcium uptake transiently increased mitochondrial production of reactive oxygen species (ROS), which locally activated the guanosine triphosphatase (GTPase) RhoA, triggering F-actin accumulation at the site of injury and facilitating membrane repair. We have evidence from our laboratory that filamin A, an actin binding protein, immunoprecipitates dysferlin, beta-1 integrin, and gamma-actin. Mitochondrial fragmentation (Figure 9A) has furthermore been implicated in FKBP8-dependent mitophagy under stress conditions [65], placing FKBP8 and consequently dysferlin, to which it binds, at key sites for membrane repair. FKBP8/38 agonists can reduce fat-induced hyperlipidemia [66], with hyperlipidemia being a key feature of dysferlinopathy in LGMD2B/LGMDR2.

We suggest that dysferlin could represent a molecular bridge between PDCD6 and FKBP8, dependent on [Ca^2+^], given that the motif positions on dysferlin utilized in binding PDCD6 and FKBP8 do not overlap (Figure 9A). Release of annexin A2 from PDCD6 by elevation of calcium accompanying repair would permit interaction of PDCD6 with interacting protein PDCD6IP, an interaction which is positively calcium-dependent [67]. PDCD6IP has been cited as a member of a membrane repair complex with annexins [68]. Annexin A2 would then be available to interact with dysferlin, positively dependent on calcium concentration. PDCD6IP, at the same time as interacting with PDCD6, regulates/enhances cofilin-mediated actin de-polymerization/turnover/fragmentation in conjunction with destrin (ADF), interacting with many forms of actin: α1-actin, F-actin capping protein subunit α2 (CAPZA2), and α1-actin, the latter interacting with filamin A, completing the cycle of interacting proteins (Figure 9A), overlapping the action of PDCD6IP. Calcium-dependent mitochondrial fragmentation enables localized signaling required for cell repair [64] and mitochondrial fragmentation depends on mitochondrial FKBP8 [65]. Whether mitochondrial cell membrane is utilized for patch repair is an interesting question, the answer unknown at present.

## 4. Materials and Methods

### 4.1. Synthesis of Proteins for Binding Studies

Oligonucleotide primers designed with Accelrys software (San Diego, CA, USA) were used to produce cDNAs for proteins in the present study (Tables S1 and S2), based on corresponding sequences in the native proteins that were considered most likely to exhibit binding properties. Restriction site regions which allowed insertion into the polycloning site of pRSET-A vector are underlined in Table S1. PCR reactions with rat skeletal muscle cDNA obtained from Amsbio (Cambridge, MA, USA) were carried out in either 25 µL or 50 µL reaction volumes containing BD Advantage 2 polymerase mix (BD Biosciences-Clontech, San Jose, CA, USA). The temperature profile of the PCR reactions was 95 °C for 3 min, 40 cycles of 95 °C for 45 s, 60 °C for 30 s, and 72 °C for 1.5 min, followed by a 10 min extension at 72 °C. Appropriately sized PCR products were sliced from low-melting-point agarose gels and the DNA was extracted using a Qiaex II PCR Purification Kit (Qiagen, Valencia, CA, USA) and sequence-verified (Figure 1 and Table S1). cDNA of dysferlin canonical C2A [22] and C2F regions (Figure 1) and putative dysferlin binding partners were ligated into hexahistidine fusion-tag pRSET-A (Life Technologies) vectors and cloned in E. coli JM 109 cells (Promega) [10,17,30,69]. Restriction enzyme analysis of minipreps accompanied by nucleotide sequencing indicated plasmids containing the desired in-frame sequences used to transform E. coli BL21 (DE3) cells. Cells were plated, and clones that showed robust expression were cultured in large scale (100–500-mL). Resulting cultures containing the vectors were induced with 1 mM isopropyl-β-D-thiogalactoside (IPTG) and incubated for 3–5 h at 37 °C. The cultured cells were centrifuged and the cell pellets disrupted by lysozyme with inclusion of protease inhibitors and DNase (Sigma-Aldrich). Ultrasonication was performed 5 times with a ½-inch sonicator tip in 10 sec pulses with 1 min gaps (Fisher Sonic Dismembrator Model 500). Following centrifugation at 20,818× *g* for 30 min, the clear supernatant was mixed with 0.5 mL of equilibrated Talon cobalt affinity resin (Takara Bio, Kusatsu, Shiga, Japan) containing protease inhibitors and incubated with shaking overnight at 4 °C. The resin was washed 3–5 times with buffer containing 10–20 mM imidazole to remove nonspecific binding proteins (His TALON buffer, Takara Bio), and bound proteins were eluted with 100 mM imidazole and centrifuged. The supernatant containing the fusion proteins was then dialyzed with Amicon Ultra-0.5 centrifugal filter (Amicon/Millipore-Sigma, Burlington, MA, USA) to remove SPR-interfering imidazole. Phosphate-buffered saline containing 0.1% Tween (PBST) with protease inhibitors was used as an exchange buffer (3–5 rounds of washing). All of the protein constructs were soluble in buffer containing 0.1% Tween 20, characterized as maintaining native protein conformations [70]. Regarding solubility, it should also be noted that our protein constructs were less than full length and thus significantly smaller than the native proteins (for example, the AHNAK construct was approximately 55 kDa, compared to its 700 kDa size). After ultrafiltration, the protein concentration was determined with a Qubit fluorescence system (Invitrogen/Life Technologies, Waltham, MA, USA). The purity of the proteins was visualized with chemiluminescence detection on Western blots (Figure S1). The primary antibodies for these Westerns (Anti-Xpress and Invitrogen Qiagen Anti-His HRP conjugates) used to detect synthesized peptides would not miss contaminants (if present), given that they targeted sequence of pRSET-A vector to which the synthesized peptides covalently joined, with the peptides recognized on Westerns by predicted molecular mass of the fusion proteins. Additionally, we detected dysferlin cC2A construct 1 with “Romeo anti-dysferlin” (ABclonal Technology) targeting specific sequence.

### 4.2. Western Blot Analysis

Affinity-purified fusion proteins were electrophoresed on a 4–12% NuPAGE gel, transferred to a PVDF (polyvinylidene fluoride) membrane, blocked with 4% nonfat milk at 4 °C overnight, and incubated in Anti-Xpress monoclonal antibody (Invitrogen, 1:1000), anti-RGS-his-HRP monoclonal antibody (Qiagen, 1:1000), or “Romeo” anti-dysferlin monoclonal antibody (ABclonal Technology, Woburn, MA, USA, < 1:500) overnight at 4 °C. After three 10 min washes with phosphate-buffered saline containing 0.1% Tween 20, the blots were incubated in HRP-conjugated bovine anti-mouse IgG secondary antibody (Santa Cruz Biotechnology, Dallas, TX, USA) (1:2500), HRP-conjugated donkey anti-mouse secondary antibody, or HRP-conjugated donkey anti-rabbit secondary antibody (Invitrogen) overnight at 4 °C. The proteins were detected with chemiluminescence (Pierce™ ECL Western Blotting Substrate; ThermoFisher Scientific, Grand Island, NY, USA).

### 4.3. Surface Plasmon Resonance (SPR) Binding Analysis

For SPR experiments, we used a GE Biacore 3000 instrument (Biacore, GE Healthcare, Piscataway, NJ, USA). Purified dysferlin domains and target protein candidates were utilized in direct binding assays at 25 °C as previously described [15,16,30,69,71]. All proteins for SPR were synthesized as pRSET-A constructs. The chosen affinity-purified hexahistidine-tagged fusion protein ligand was immobilized on a CM5 (Biacore) chip using an amine coupling protocol [14,15,16]. Each ligand response was compared to a reference for subtraction of nonspecific binding. For a typical analysis, a ligand concentration yielding 500–3000 response units (RUs) was employed. After establishing association and dissociation conditions, kinetic binding analyses were conducted from interactions of ligand and analyte over a range of analyte concentrations, typically 0–320 nM, including reversal of ligand and analyte. After analysis, the chip was regenerated with 1 M NaCl. Kinetic values were ascertained using BIAevaluation software version 3.0 (Biacore) with a 1:1 Langmuir binding model selected for calculations across analyte concentrations. Protein domains studied are assumed to have tertiary configurations similar to those in the complete proteins, since proteins were synthesized and analyses were performed in physiologically compatible solvents.

### 4.4. Immunohistochemistry

Rat skeletal muscle (biceps femoris), paraffin-embedded transverse and longitudinal tissue sections, were obtained from Zyagen (RP-102; San Diego, CA, USA). Immunoreactivity was visualized with the avidin–biotin–complex peroxidase method (ABC Elite protocol, Vector, Burlingame, CA, USA) with 3,3′-diaminobenzidine (DAB) serving as chromogen (Bio-Genex, San Ramon, CA, USA). Four- to five-micrometer deparaffinized sections were sequentially incubated in 0.1% sodium borohydride, 5 mM glycine in phosphate-buffered saline (PBS) for 45 min, 3% H_2_O_2_ in tap water for 5 min, 2% normal serum (corresponding to the species in which the secondary antibody was raised) in PBS, and primary antibody at 4 °C for 12–16 h [72], followed by biotinylated donkey anti-mouse, anti-rabbit or anti-goat secondary antibodies for 30 min at room temperature. Immunostaining with DAB was examined with a Leitz Diaplan microscope (Leitz, Wetzlar, Germany) and photographed with an Olympus OM-4T camera, and the negatives were digitized at 300–600 dpi. Individual DAB negative controls, corresponding to omission of primary antibody with inclusion of secondary antibodies, monoclonal and polyclonal donkey anti-rabbit IgG, polyclonal donkey anti-mouse IgG, and polyclonal donkey anti-goat IgG, can be found in Figure S2.

Colocalization of protein immunofluorescence was examined with confocal microscopy immunofluorescence Z-stacks using a Zeiss LSM 780 instrument at 63× magnification and 0.6–1.0 μm slices (Zen 2.1 software). Negative controls (Figure S2) included omission of primary antibody and/or replacement of the primary antibody by purified IgG for the species that was used to raise the primary. In Z-stack fluorescence confocal microscopy, fluorescence signal was determined for experimental localizations with gains of individual channels separately set, yielding no immunofluorescence background for negative controls, followed by experimental measurements at those settings.

Primary antibodies included NCL-Hamlet mouse monoclonal for dysferlin targeting synthetic peptide aa 1999–2016 of human dysferlin and crossing to rat dysferlin (Leica, Buffalo Grove, IL, USA); mouse monoclonal antibody targeting human annexin A2 339-aa fusion protein crossing to rat annexin A2 (66035-1-Ig, Clone No. 1C1E12, Proteintech); annexin A2 affinity-purified polyclonal goat antibody targeting human annexin A2 carboxy terminus crossing to rat (SC-1924, Santa Cruz Biotechnology, Dallas, TX, USA); PDCD6 rabbit polyclonal antibody targeting human amino terminus first 191 aa crossing to rat (12303-1-AP, Proteintech); FKBP8/38 rabbit polyclonal antibody against mouse FKBP8/38 (ab24450, ABCAM, Cambridge, MA, USA); FKBP8 rabbit polyclonal antibody targeting first 355 aa human FKBP8 crossing to mouse (11173-1-AP, Proteintech, Rosemont, IL, USA); and TMC1 rabbit polyclonal antibody targeting mouse TMC1 amino terminus (ABN 1649, Millipore/Sigma, St. Louis, MO, USA).

The primary antibodies were coupled to Molecular Probes/Invitrogen (Carlsbad, CA, USA) secondary antibodies donkey anti-mouse IgG (H + L) highly cross-absorbed secondary antibody Alexa Fluor 568, and donkey anti-rabbit IgG (H + L) highly cross-absorbed secondary antibody Alexa Fluor 488.

## 5. Conclusions

Taken together, the results of the experiments presented, in addition to those from the literature, suggest a model of repair (see Figure 9). The finding that dysferlin C2 heterodomain interactions display negative calcium dependence similar to that of the related ferlin, otoferlin [10], suggests a similarity of mechanism: otoferlin is hypothesized to shift from interactions between its own C2 domains to C2 binding to soluble NSF attachment protein receptors (SNAREs) in the hair cell synaptic complex, as the calcium concentration increases during depolarization. Thus, dysferlin, similar to otoferlin, would assume a more compact structure at low calcium and less compact at increased calcium, facilitating binding to coordinating proteins. With injury and elevation of intracellular Ca^2+^ concentration, membrane repair would be initiated via differential direct binding of dysferlin C2 domains and dysferlin protein complex partners, and consequent readjustment of protein binding partners toward those interactions that are positively dependent on calcium. However, unlike the findings for otoferlin, these dysferlin repair complex proteins for elevated calcium reflect direct interactions primarily of the dysferlin cC2A domain. In the present model, annexin A1, calpain-3, mitsugumin-53, and affixin, along with dysferlin, actin, and β1-integrin, would first respond to calcium influx resulting from injury, and caveolin-3, syntaxin-4, and AHNAK1 would interact during resting conditions before calcium entry, thus serving steady-state maintenance. Dysferlin would serve as a bridge linking PDCD6 and FKBP8 (Figure 9A,B), subsequently linking the sarcolemma with the subsarcolemmal population of mitochondria that would be first impacted by calcium overload associated with injury.

## Figures and Tables

**Table 1:** Binding partners of dysferlin C2 domains associated with repair processes in skeletal muscle as determined by SPR analysis.

Binding Partners	K_D_ ± SEM	(n)	Ca^2+^ Dependence
Dysf C2A—Annexin A1	2.5 ± 0.8 × 10^−8^	(3)	+++
Dysf C2F—Annexin A1	3.3 ± 0.3 × 10^−7^	(3)	+
Dysf C2A—Calpain-3	1.2 ± 0.4 × 10^−6^	(3)	+++
Dysf C2F—Calpain-3	2.2 ± 0.6 × 10^−9^	(3)	++
Dysf C2A—Mitsugumin-53	4.7 ± 0.7 × 10^−7^	(3)	+++
Dysf C2F—Mitsugumin-53	nd		0
Dysf C2A—Affixin	4.1 ± 1.1 × 10^−5^	(5)	++
Dysf C2F—Affixin	4.0 ± 1.0 × 10^−7^	(4)	+++
Dysf C2A—Caveolin-3	3.3 ± 0.6 × 10^−8^	(3)	+
Dysf C2F—Caveolin-3	1.1 ± 0.5 × 10^−7^	(3)	−
Dysf C2A—Syntaxin-4	4.4 ± 1.3 × 10^−6^	(3)	+
Dysf C2F—Syntaxin-4	1.3 ± 0.4 × 10^−7^	(3)	−
Dysf C2A—AHNAK1	3.8 ± 1.6 × 10^−7^	(3)	+
Dysf C2F—AHNAK1	nd		0

## Data Availability

Essentially all of the data associated with this paper can be found within the paper itself. Cited nucleotide and protein sequences are available from GenBank, National Center for Biotechnology Information, National Library of Medicine, USA.

## Supplementary Materials

The following supporting information can be downloaded at: https://www.mdpi.com/article/10.3390/ijms24054707/s1.
