# Supplementary material for: Analysis of Dysferlin Direct Interactions with Putative Repair Proteins Links Apoptotic Signaling to Ca2+ Elevation via PDCD6 and FKBP8
Source: Int J Mol Sci. 2023 Feb 28;24(5):4707. doi: 10.3390/ijms24054707 (PMC10002499; doi:10.3390/ijms24054707)
Supplement: Supplementary file 1 [file ijms-24-04707-s001.zip › ijms-2123520-supplementary.pdf]

International Journal of *Molecular Sciences*

MDPI

Supplementary Materials

Analysis of Dysferlin Direct Interactions with Putative Repair Proteins Links Apoptotic Signaling to Ca^{2+} Elevation via PDCD6 and FKBP8

Dennis G. Drescher [1,2,*], Marian J. Drescher [1], Dakshnamurthy Selvakumar [1], and Neeraja P. Annam [1]

1 Laboratory of Bio-otology, Department of Otolaryngology, Wayne State University School of Medicine, Detroit, MI 48201, USA;
2 Department of Biochemistry, Microbiology and Immunology, Wayne State University School of Medicine, Detroit, MI 48201, USA
* Correspondence: ddresche@med.wayne.edu

Table S1. PCR primers used in the present studies with pRSET-A.

Construct	GenBank Accession Number	Primers	Restriction Sites
Dysferlin cC2A (canonical)	XM_006236773.4	USa: GCTAGGATCCATCAGCGATGCTTACTGC<br>USb GCTAGGATCCATGCTGCGAGTCTTCAT<br>USc: GCTAGGATCCATGCTGCGAGTCTTCATT<br>DSa: TCTAGAATTCTCATGGGAACAAGGGCGCAGC<br>DSb: TCTAGAATTCTCATGGGAACAAGGG<br>DSc: TCTAGAATTCTCACCCAGAATATCTCGT	5′ BamHI<br>5′ BamHI<br>5′ BamHI<br>3′ EcoRI<br>3′ EcoRI<br>3′ EcoR
Dysferlin C2B	XM_006236773.4	US: TCTAGGATCCCTGTCAGACAAGCCACAGGAC<br>DS: GCTAGAATTCTCAAGAGAAGTCATCGGGATC	5′ BamHI<br>3′ EcoRI
Dysferlin C2C	XM_006236773.4	US: TCTAGGATCCCTTCGTGGTGCCCATTTTTGC<br>DS: GCTAGAATTCTCACAACAGAAGCCTGCCCCT	5′ BamHI<br>3′ EcoRI
Dysferlin C2D	XM_006236773.4	US: TCTAGGATCCTATGGGAACCGCTACCATCTG<br>DS: GCTAGAATTCTCAGATGGCTGGCTTCTCTCT	5′ BamHI<br>3′ EcoRI
Dysferlin C2DE	XM_006236773.4	US: GCCGGATCCGCCATCCATCACATCCCTGGCT<br>DS: TGCGAATTCTTACTGGGGACCCTGAGCAGC	5′ BamHI<br>3′ EcoRI
Dysferlin C2E	XM_006236773.4	US: TCTAGGATCCCAGGAATGCTTGGTCCGGATT<br>DS: GCTAGAATTCTCAGCAGAAGAGGTGGAGGAG	5′ BamHI<br>3′ EcoRI
Dysferlin C2F	XM_006236773.4	US: TGCGGATCCCTGCGTTGTATTATTTGGA<br>DS: TGCGAATTCTCACAAGGAGCACTTCTCAGC	5′ BamHI<br>3′ EcoRI
Dysferlin COOH	XM_006236773.4	US: TGGGGATCCAGTGAACATGAGGAGCGGCCT<br>DS: TGCGAATTCTCACACCAGCTTCATGGCAGCATAGTTC	5′ BamHI<br>3′ EcoRI
Annexin A1	NM_012904	US: GCTAGGATCCATGGCAATGGTATCAGAA<br>DS: TCTAGAATTCTTAGTTTCCTCCACACAGAGC	5′ BamHI<br>3′ EcoRI
Annexin A2	XM_039082021	US: GCCGGATCCATGTCTACTGTCCACGAA<br>DS: TGCGAATTCTTACAGGCCTAACATCACGGT	5′ BamHI<br>3′ EcoRI
Mitsugumin 53	NM_001077675.1	US: GCTACTCGAGATGTCGACTGCACCAGGCCTTTTG<br>DS: TCTAGAATTCTCAGGCCTGCTCACTGTCTGGCCCCAC	5′ XhoI<br>3′ EcoRI
Calpain 3	NM_017117.2	US: GCTACTCGAGGTGGACCAGGAGGCAGAGGAG<br>DS: TCTAGAATTCTCAGGCATACATGGTAAGCTGCAG	5′ XhoI<br>3′ EcoRI
Affixin	NM_001134780.1	US: GCTACTCGAGAGCGCCATCAATTCTCCAATG<br>DS: TCTAGAATTCTCACAGGCCTTCCCGCTTCCGGAC	5′ XhoI<br>3′ EcoRI
Caveolin 3	NM_019155.2	US: GCTACTCGAGATGATGACCGAAGAGCACACA<br>DS: TCTAGAATTCTTAGCCTTCCCTTCGCAGCACCAC	5′ XhoI<br>3′ EcoRI
Syntaxin 4	L20821.1	US: GCTAGGATCCATGCGCGACAGGACCCAT<br>DS: TCTAGAATTCTCAGACCTTTTTCTTCCTCGC	5′ BamHI<br>3′ EcoRI
AHNAK 1	NM_001398671.1	US: GCTACTCGAGTCTGGAGACATCAAGTGTCCT<br>DS: TCTAGAATTCCCTACTCTTTCTTTGTGGAAACTGA	5′ XhoI<br>3′ EcoRI
PDCD6	NM_001107452.1	US: GCCCTCGAGATGGCTGCCTACTCCTACCGCCCAGGCCCA<br>DS: TGCCTGCAGTTAACAATGCTGAAAACCATGGAGAGATA	5′ XhoI<br>3′ PstI
FKBP8	NM_001037180.1	US: GCCGGATCCCTGGAAGTCACCCTGAAGACA<br>DS: TGCGAATTCTCAGTTCCTGGCAGCAATGA	5′ BamHI<br>3′ EcoRI

Primers are based on rat sequences. US, upstream primers; DS, downstream primers. Primer sequences are written 5′ to 3′ with designated restriction sites underlined.

Table S2. Protein constructs used in the present studies.

Construct	Size	GenBank Accession Number	Sequence
Annexin-A1	108 aa	NP_037036.1	MAMVSEFLKQACYIEKQEQEYVQAVKSYKGGPGSAVSPYPSFNPSSDVAALHKA<br>IMVKGVDEATIIDILTKRTNAQRQQIKAAYLQETGKPLDETLKKALTGHLEEVV
Annexin-A2	102 aa	XP_038937949.1	MSTVHEILCKLSLEGDHSTPPSAYGSVKPYTNFDAERDALNIETAIKTKGVDEVTI<br>VNILTNRSNAQRQDIAFAYQRRTKKELPSAMKSALSGHLETVMLGL
Mitsugumin-53	477 aa	NP_001071143.1	MSTAPGLLRQELSCPLCLQLFDAPVTAECGHSFCRACLIRVAGEPADDGTVACPC<br>CQASTRPQALSTNLQLARLVEGLAQVPQGHCEEHLDPLSIYCEQDRTLVCGVCAS<br>LGSHRGHRLLPAAEAHARLKTQLPQQKAQLQEACMRKEKSVAVLEHQLVEVEE<br>TVRQFRGAVGEQLGKMRMFLAALESSLDREAERVRGEAGVALRRELSSLNSYLEQ<br>LRQMEKVLEEVADKPQTEFLMKFCLVTSRLQKILSESPPPARLDIQLPVISDDFKFQ<br>VWKKMFRALMPELEELTFDPSSAHPSLVVSASGRRVECSEQKAPPAGEDTCQFDK<br>TVAVVAKQLLSQGEHYWEVEVGDKPRWALGVMAADASRRGRLHAVPSQGLWL<br>LGLRDGKILEAHVEAKEPRALRTPERPPARIGLYLSFADGVLTFYDASNTDALTPLF<br>SFHERLPGPVYPMFDVCWHDKGKNSQPLLLVGPDSEQA
Calpain-3	178 aa	NP_058813.2	VDQEAEEGKDKTGPDKQGESPQPRPGHTDQESEEQQQFRNIFRQIAGDDMEICAD<br>ELKNVLNTVVNKHKDLKTQGFTLESCRSMIALMDTDGSGRLNLQEFHHLWKKIK<br>AWQKIFKHYDTDHSGTINSYEMRNAVNDAGFHLNSQLYDIITMRYADKHMNIDF<br>DSFICCFVRLEGMFRAFHAFDKDGIIKLNVLEWLQLTMYA
Affixin	170 aa	NP_001128252.1	SAINSPMAPALVDIHPEDTQLEENEERTMIDPTSREDPKFKELVKVLLDWINDVLA<br>EERIIVKQLEEDLYDGQVLQKLLEKLAHCKLNVAEVTQSEIGQKQKLQTVLEAVQ<br>DLLRPHGWPLRWNVDSIHGKNLVSILHLLVSLAMHFRAPIHLPEHVTVQVVVVR<br>KREGL
Caveolin-3	151 aa	NP_062028.1	MMTEEHTDLEARIIKDIHCKEIDLVNRDPKNINEDIVKVDFEDVIAEPEGTYSFDGV<br>WRVSYTTFTVSKYWCYRLLSTLLGVPLALLWGFLFACISFCHIWAVVPCIKSYLIEIQ<br>CISHIYSLCIRTFCNPLFAALGQVCSNIKVVLRREG
Syntaxin-4	298 aa	AAA03046.1	MRDRTHELRQGDNISDDEDEVRVALVVHSGAARLSSPDDEFFQKVQTIRQTMAKL<br>ESKVRELEKQQVTILATPLPEESMKQGLQNLREEIKQLGREVRAQLKAIEPQKEEA<br>DENYNSVNTRMKKTQHGVLSQQFVELINKCNSMQSEYREKNVERIRRQLKITNAG<br>MVSDEELEQMLDSGQSEVFVSNILKDTQVTRQALNEISARHSEIQQLERTIRELHE<br>IFTFLATEVEMQGEMINRIEKNILSSADYVERGQEHVKIALENQKKARKKKVMIAI<br>CVSVTVLILAVIIGITITVG
AHNAK	500 aa	NP_001385600.1	SGDIKCPKVSVGTPEVSVEALEGGVKLPQMKLPQFGISTPGSDLDINIKGPQVCGE<br>LQGSGTDVNLKGPQISAPGMDFNLEGPKVRGSLGATGELKGPSIGGGLPGISIQGP<br>EGNLQMPGIKASGCDVKVSSGQISGPEIKGDLKGSGIGLHGAVPDLSVKGPSFNM<br>ASPESDFGVSLKGPKVKGGVDVSGGVSVPDINLGEGHMNVKGFGGEWKGPQVSS<br>SVNLDTTKLVGNLHFSGPKIEGDVKGGQIGLQGPGLSVSGPQGHLESGSGKVTFP<br>KMKIPKFTFSGRELTGREVGVDVNFPNVEANVQAGAGEGELEESAVKLKKSKIKM<br>PKFTFSKPKGKGGVTGSPEASVSGSKGDLKSSKASLGSLEGEAEAETSSPKGKFSLF<br>KSKKPRNRSNSFSDEREFSAPSTPTGTLEFAGGEGKGKHGKLKFGTFGGLGSKSKG<br>HYEVTGSDDEAGKLQGSGVSLASKKSRLSSSSSNDSGTKLGIQLPEVEISVSTKKE
PDCD6	180 aa	NP_001100922.1	MAAYSYRPGPGAGPGPAAGAALPDQSVLWNVFQRVDKDRSGVISDNELQQALS<br>NGTWTPFNPVTVRSIISMFDRENKAGVNFSEFTGVWKYITDWQNVFRTYDRDNS<br>GMIDKHELKQALSGFGYRLSDQFHDILIRKFDRQGRGQIAFNDFVQGCIVLRRLTD<br>IFRRYDTDQDGWIQVSY
FKBP8	217 aa	NP_001032257.1	LEVTLKTAEDGPDLEMLSGQERVALANRKRECGNAHYQRADFVLAANSYDLAIK<br>AITSNAKVDMTCEEEEELLQLKVKCLNNLAASQLKLDHYRAALRSCSQVLEHQPD<br>NIKALFRKGKVLAQQGEYSEAIPILRAALKLEPSNKTIHAELSKLVKKRAAQRSTET<br>ALYRKMLGNPSRLPAKCPGKGAWSIPWKWLFGATAVALGGVALSVVIAARN

Protein sequences shown were synthesized via pRSET-A primers.

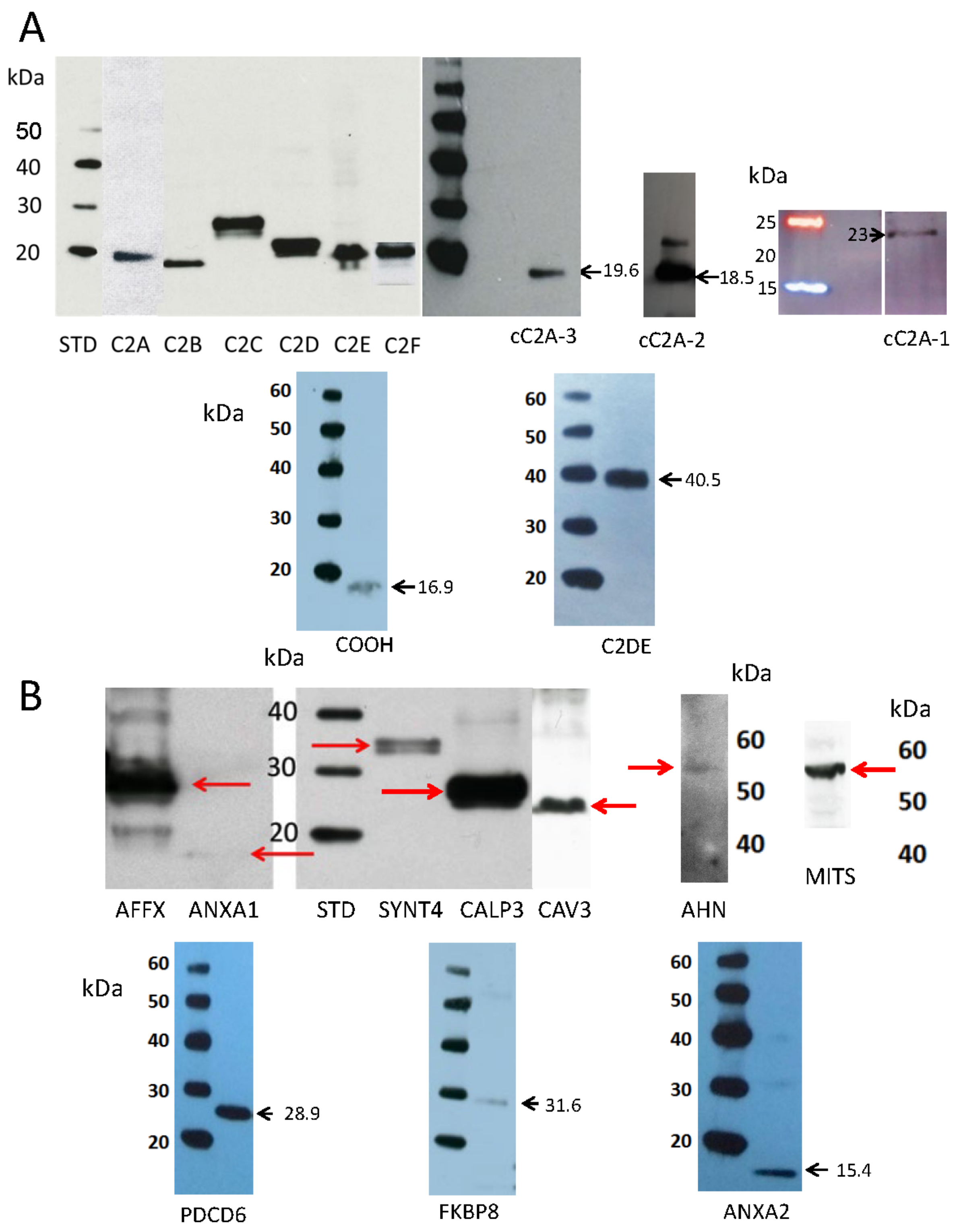

Figure S1. Western blots for pRSET-A fusion protein constructs used for SPR with mass designations including plasmid contributions. (**A**) Dysferlin C2 domain constructs. The molecular mass for cC2A (canonical) is 19.6 kDa; for C2B, 18 kDa; C2C, 24 kDa; C2D, 20 kDa; C2E, 18 kDa; C2F, 20 kDa; cC2A-3 = 19.6 kDa; cC2A-2 = 18.5 kDa; cC2A-1 = 23 kDa; COOH terminus for dysferlin = 16.9 kDa; C2DE = 40.5 kDa. (**B**) Constructs for proteins interacting with dysferlin cC2A and C2F domains: affixin (AFFX), 24 kDa; annexin A1 (ANXA1), 16 kDa; syntaxin-4 (SYNT4), 36 kDa; calpain-3 (CALP3), 28 kDa; caveolin 3 (CAV3), 22 kDa; AHNAK (AHN), 55 kDa; mitsugumin-53 (MITS), 57 kDa; PDCD6, 28.9 kDa; FKBP8, 31.6; annexin A2 (ANXA2), 15.4 kDa. Red arrows point to each protein's specific band.

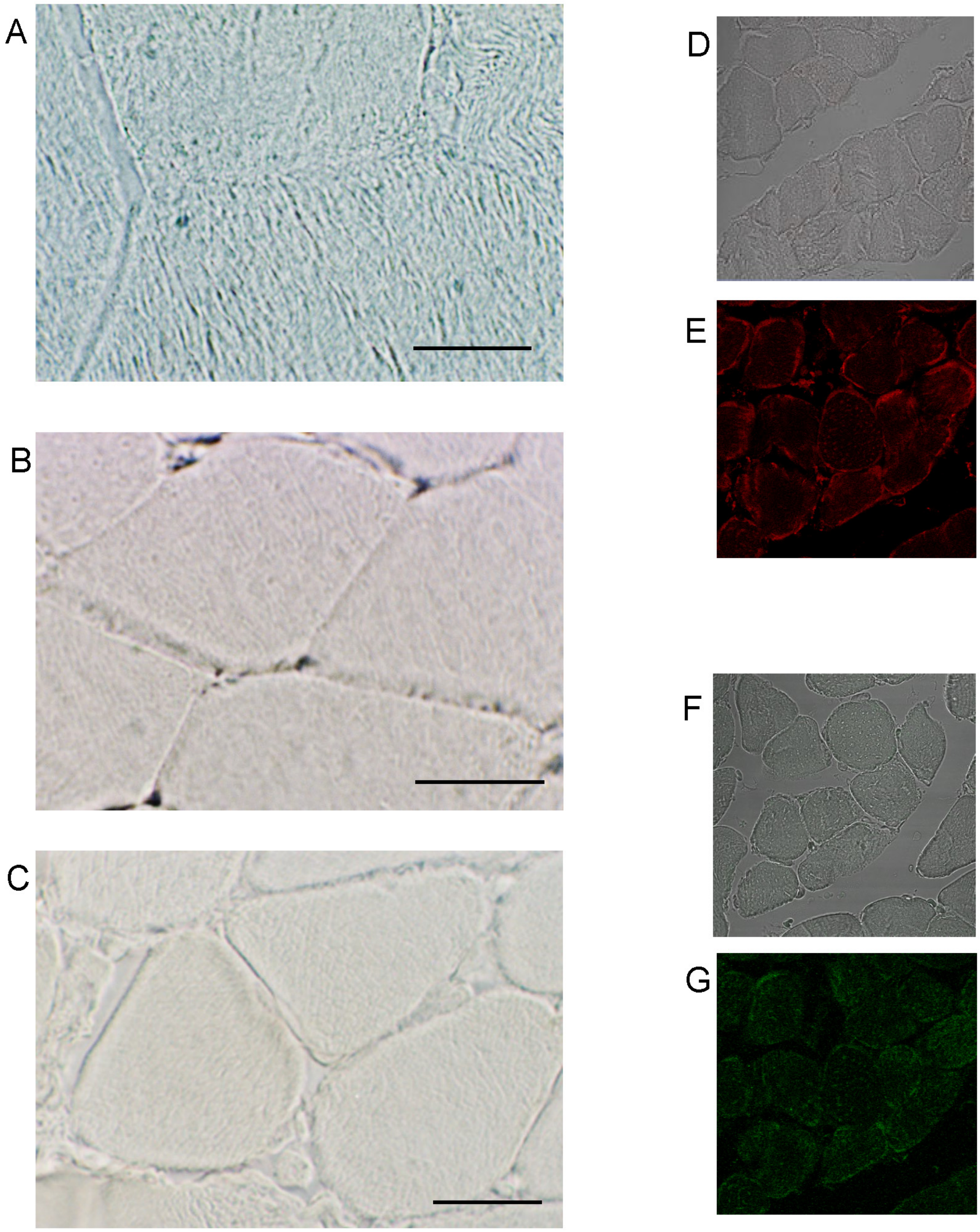

Figure S2. (**A**-**C**) Negative controls for DAB immunolocalizations. (**A**) Donkey anti-rabbit IgG polyclonal secondary antibody. Primary antibody omitted. Scale bar = 10 µm. (**B**) Donkey anti-mouse IgG polyclonal secondary antibody. Primary antibody omitted. Scale bar = 10 µm. (**C**) Donkey anti-goat IgG polyclonal secondary antibody. Primary antibody omitted. Scale bar = 10 µm. (**D**-**G**) Negative controls for immunofluorescence localizations. (**D**) Negative control (no primary antibody; secondary antibody Alexa Fluor 568 + brightfield). (**E**) Primary antibody for FKBP8, secondary antibody Alexa Fluor 568, same gain as for (**D**). (**F**) Negative control (no primary antibody; secondary antibody Alexa Fluor 488 + brightfield. (**G**) Primary antibody for mitochondrial calcium uniporter (MCU), a mitochondria inner-membrane protein (Alomone Labs, Jerusalem, Israel); secondary antibody Alexa Fluor 488, same gain as for (**F**)
